# Changes in anemia and anthropometry during adolescence predict learning outcomes: findings from a 3-year longitudinal study in India

**DOI:** 10.1093/ajcn/nqac028

**Published:** 2022-02-04

**Authors:** Phuong H Nguyen, Monika Walia, Anjali Pant, Purnima Menon, Samuel Scott

**Affiliations:** Poverty, Health and Nutrition Division, International Food Policy Research Institute, Washington, DC, USA; Poverty, Health and Nutrition Division, International Food Policy Research Institute, New Delhi, India; Poverty, Health and Nutrition Division, International Food Policy Research Institute, New Delhi, India; Poverty, Health and Nutrition Division, International Food Policy Research Institute, New Delhi, India; Poverty, Health and Nutrition Division, International Food Policy Research Institute, New Delhi, India

**Keywords:** adolescent, anemia, thinness, stunting, reading proficiency, math proficiency, India

## Abstract

**Background:**

Anemia and poor physical growth during adolescence have far-ranging consequences, but limited longitudinal evidence exists on how changes in these factors relate to changes in learning skills as adolescents mature.

**Objectives:**

We examined the association between changes in anemia and physical growth during adolescence and learning outcomes.

**Methods:**

We used longitudinal data from the Understanding the Lives of Adolescents and Young Adults (UDAYA) project, which surveyed adolescents aged 10–19 y in northern India in 2015–2016 and 2018–2019 (*n* = 5963). We used multilevel mixed-effects logistic regression models to examine associations between changes in anemia/thinness/stunting status (4 groups: never, improved, new, and persistent) and reading (ability to read a story) and math proficiency (ability to solve division problems) at follow-up.

**Results:**

Persistent anemia and stunting were higher among girls than among boys (46% compared with 8% and 37% compared with 14%, respectively), but persistent thinness was lower (7% compared with 16%). Improvement in anemia, thinness, and stunting was 1.4–1.7 times higher among boys than among girls. Boys who were anemic in both waves were 74% [adjusted odds ratio (AOR): 0.26; 95% CI: 0.12, 0.59] and 65% (AOR: 0.35; 95% CI: 0.16, 0.76) less likely to be able to read a story and solve division problems, respectively, than boys who were nonanemic in both waves. Persistent thinness in boys was negatively associated with both reading (AOR: 0.37; 95% CI: 0.21, 0.66) and math proficiency (AOR: 0.27; 95% CI: 0.16, 0.46). Persistent stunting contributed to lower reading and math proficiency in boys and girls (AORs: 0.29–0.46). Boys whose anemia or thinness status improved and girls whose stunting status improved had similar learning skills at follow-up as those who were never anemic/thin/stunted.

**Conclusions:**

Persistent anemia, thinness, and short stature during adolescence were associated with poor learning. Programs targeted at adolescents should contribute to nurturing environments that foster healthy growth and learning.

## Introduction

Globally, there are an estimated 1.8 billion adolescents, with 90% residing in low- and middle-income countries and 1 in 5 residing in India (1). Poor nutrition during this period is common, with an estimated 1 in 4 adolescents (∼430 million globally) suffering from anemia and 10% underweight (2). In India, the 2006 and 2016 rounds of the National Family Health Survey (NFHS) showed no anemia reduction among female (54%) or male (29%) adolescents aged 15–19 y (3, 4) and stunting affects one-third of adolescents in this population (5).

Despite the widespread nature of undernutrition during adolescence, investments to promote optimal health and development for this age range have been insufficient (6), with the first 1000 d taking center stage in the global human development agenda. An estimated 95% of child and adolescent health and development research between 2004 and 2017 focused on children under the age of 5 y (6). However, physical and mental development takes ≥2 decades—8000 d beginning with conception—and includes childhood and adolescence, both periods of physical growth, restructuring of the brain, and socio-behavioral change (6). Addressing the needs of adolescents can contribute to progress toward the UN's Sustainable Development Goals, all of which directly or indirectly relate to adolescent health, development, or well-being.

Poor environments leading to compromised physical growth during adolescence can result in far-ranging and intergenerational consequences. Poor physical growth among adolescent girls adversely affects their health and development, and is associated with poor birth outcomes such as low birth weight, preterm birth, stillbirth, and an increased risk of neonatal mortality (7). Adolescent physical growth and anemia have also been associated with human capital outcomes, with cross-sectional studies showing negative associations of stunting, underweight, low BMI, and low iron status/anemia with cognitive skills, school attendance, school performance, and grade attainment (8–11). Longitudinal evidence reports associations between improved growth during the first few years of life and later development outcomes (12), but less is known about how changes in anthropometry or anemia status during adolescence are related to learning outcomes. The Young Lives studies in Ethiopia, India, Peru, and Vietnam showed that improved physical growth in early and middle childhood was associated with more schooling and higher math, reading, and receptive vocabulary scores across countries (13–15), although others have questioned the methods underlying these findings (16). Two studies in India found that iron interventions led to improvements in cognitive abilities (17, 18). A recent study, using Understanding the Lives of Adolescents and Young Adults (UDAYA) data, showed that receiving iron and folic acid supplementation was positively associated with learning outcomes, whereas dietary diversity was positively associated with height-for-age *z*-score (HAZ), math proficiency, and reduced risk of dropout (19). A better understanding of the association between multiple types of growth failure and learning outcomes is needed as countries make decisions about where to focus their human capital investments.

Using longitudinal data on female and male adolescents 10–19 y of age in northern India, our study aimed to examine the relation between physical growth failure (thinness and stunting), anemia, and learning skills (reading and math proficiency) during adolescence. We aimed to answer 3 key research questions: *1*) What is the prevalence of growth failure and anemia in this population and what percentage of adolescents can read a story and solve division problems? *2*) Is persistent growth failure or anemia during adolescence related to poorer learning outcomes? *3*) Do adolescents whose physical growth or anemia status improves during adolescence show similar learning abilities at follow-up as their peers who were not short, thin, or anemic at either survey wave?

## Methods

### Data source and study population

We used longitudinal data from the UDAYA project which were collected in 2 Indian states, Uttar Pradesh and Bihar, by the Population Council under the guidance of the Ministry of Health and Family Welfare, Government of India (20, 21). The project was designed to provide insights on changes that adolescents undergo as they transition from adolescence to adulthood. Full details on study design, sampling, survey instruments, and data collection can be found elsewhere (20, 21). Briefly, 150 primary sampling units (PSUs)—villages in rural areas and census wards in urban areas—were selected, and a multistage systematic sampling design was adopted within each residential area. First, villages or wards were selected systematically from the stratified list for an urban or rural area with selection probability proportional to size. This was followed by household selection with equal probability from the household listing and random selection of 3 respondents maximum within each selected household.

The UDAYA project followed a panel of 16,929 adolescents in 2015–2016 (wave 1) when they were 10–19 y old and again in 2018–2019 (wave 2) when they were 13–22 y old (20, 21). As per the sampling strategy, UDAYA specifically sampled both unmarried and married girls but did not sample married boys separately. From the larger cohort, anthropometric and biomarker data were collected from all adolescents 10–14 y old, and in a randomly selected 24% sample of adolescents 15–19 y old, after obtaining appropriate assent and consent forms. This resulted in a subsample of 7797 adolescents in 2015–2016. This group was reassessed in 2018–2019 and had a follow-up rate of 81%, with 3% loss due to migration or parent refusal and 16% loss due to participant refusal or nonavailability at home. The final analytical sample for the current article comprised 5963 adolescents (2284 boys and 3679 girls) who were surveyed in both waves and for whom data on either hemoglobin (Hb) or anthropometric measures were available (**Supplemental Figure 1**).

### Measures

#### Outcomes

**Supplemental Table 1** defines all variables used in the analysis. Our key outcomes of interest were reading and math proficiency at wave 2, which were assessed using the Annual Status of Education Report (ASER) tools (22). These tools show high reliability and validity in India (23), have been used in a nationwide survey since 2005, and are widely used in the South Asian region to assess progress in basic learning skills (24, 25). The ASER tool was administered following standard procedures by trained enumerators who had extensive training including classroom sessions, group activities, field practice, and a quiz. Data collection was carried out in households to ensure that all adolescents (whether enrolled in school or not) were included in the survey. Field supervision and phone and desk recheck procedures were used for quality control of the assessment. Reading proficiency was measured as the ability to read in the Hindi language on 4 levels: ability to recognize letters, read words, read a short paragraph, and read a story (standard II level, i.e., children aged 7–8 y). Math proficiency was also measured on 4 levels: ability to recognize single-digit numbers, recognize double-digit numbers, solve 2-digit subtraction problems, and solve 3-digit division problems. Our outcomes of interest were dummy variables for ability to read a story (reading outcome) and ability to solve a division problem (math outcome).

#### Explanatory variables

Our key explanatory variables were anemia, thinness, and stunting. Hb concentrations were measured from capillary blood samples by trained health investigators using a portable HemoCue Hb 201+ instrument (20, 21). Anemia was defined as altitude-adjusted Hb <115 g/L for boys and girls 10–11 y old, Hb <120 g/L for boys 12–14 y old and girls 12–19 y old, and Hb <130 g/L for boys 15–19 y old as per the WHO guidelines (26).

Adolescent weight and height were measured by trained and standardized field staff using standard methods. Weight was measured using a SECA 874 electronic scale with precision to the nearest 100 g and height was measured using a SECA 213 stadiometer with an accuracy level of 0.1 cm. The height and weight measurements were used to calculate HAZs and BMI-for-age *z*-scores (BMIZs) according to the WHO growth reference (27). Stunting and thinness were defined as HAZ and BMIZ < −2 SDs from the reference, respectively.

We characterized adolescents into 4 groups based on their anemia and physical growth status in waves 1 and 2; for example, never anemic (e.g., not anemic in waves 1 and 2), improved (e.g., anemic in wave 1 but not anemic in wave 2), newly anemic (e.g., not anemic in wave 1 but anemic in wave 2), and persistently anemic (e.g., anemic in both waves 1 and 2). Similar groups categorizing change in physical growth over time were created for thinness and stunting.

#### Confounding factors

Variables that could potentially confound the association between anemia/physical growth and learning outcomes included demographic, socioeconomic, and environmental factors. Demographic factors included age, religion (Hindu and non-Hindu), caste (scheduled caste/tribe, other backward classes, and others), currently attending school, marital status, mother's education attainment (highest level of school completed), and household wealth quintile. Data on household assets, ownership of selected durable goods, means of transportation, and access to amenities such as clean cooking fuel and electricity were used to create a wealth index score, which was then divided into 5 quintiles. Environmental factors included the place of residence (rural/urban), household's access to an improved toilet facility and improved source of drinking water, type of school where the respondent last received an education (government or private), exposure to mass media (exposed either every day or at least once a week to content on 3 out of 5 mass media sources: television, radio, newspaper/magazine/books, own mobile, Internet), and ever use of any social media platform such as Facebook or Twitter.

### Statistical analysis

We first compared baseline characteristics of study participants in the final analytic sample with those lost to follow-up using Student's *t*-test or the chi-square test. We analyzed associations between the predictors and learning outcomes separately for girls and boys. The weighted prevalence of outcomes and physical growth/anemia by age and gender over time were visualized using line charts. Multilevel mixed-effects logistic regression models were used to examine associations of changes in predictors between waves 1 and 2 (predictors) with reading and math proficiency at wave 2. The fitted multilevel models included appropriate subsample weights and 2 levels of variations, level 1 being individual and level 2 being PSU as a random effect to adjust for clustering (28). All models adjusted for demographic, birth history for girls (currently pregnant or ever gave birth) and number of siblings, socioeconomic, and environmental factors (which are often associated with anemia/physical growth and learning) to minimize confounding effects. Results from regression were presented in the form of both unadjusted ORs and adjusted ORs (AORs) and their 95% CIs. To offer an example of how to interpret the AOR, an AOR of 0.75 would be interpreted as the odds of an outcome (in our case, the ability to read a story or solve division problems) being 25% lower in the group of interest than in the reference group, after adjusting for confounding factors. All analyses were conducted in Stata software version 16 (StataCorp LLC).

### Ethics statement

This study is based on a secondary data analysis of project UDAYA data, which are publicly available, fully anonymized data sets that can be downloaded from https://dataverse.harvard.edu/dataset.xhtml?persistentId=doi:10.7910/DVN/ZJPKW5. Data collection for the primary study was approved by the institutional review board of the Population Council. For participation in the survey, consent was taken from each individual, and for unmarried adolescents aged 10–17 y consent was also taken from a parent or guardian. No additional ethics approval was required for use of the publicly available UDAYA data.

## Results

### Sample characteristics

Adolescent boys and girls were ∼13 y and ∼16 y old, respectively, during wave 1 of the survey (**Table 1**). As per UDAYA's sampling strategy (20, 21), around half of the girls were married. On average, boys and girls had completed 8 y of schooling in wave 2 but their mothers had only completed 2 y of schooling. At wave 1, 91% of boys attended school compared with 51% of girls. School attendance dropped by 16–19 percentage points (pp) among both genders between the 2 survey waves. Compared with boys, fewer girls attended private schools. Most adolescents (>80%) lived in rural, Hindu, backward caste households and almost all (∼97%) had access to an improved source of drinking water. Access to an improved toilet facility improved by 20–30 pp between the 2 survey waves. Exposure to mass media and use of social media increased over time; however, the levels of exposure and use were much lower among girls than among boys. Among the subsample with anthropometric and/or biomarker data, the analytical sample was similar in terms of baseline characteristics to those with missing data (**Supplemental Table 2**).

**TABLE 1:** Demographic, health, social, and environmental characteristics of Indian adolescents over time, Understanding the Lives of Adolescents and Young Adults (UDAYA) data 2015–2016 and 2018–2019^1^

	Wave 1: 2015–2016		Wave 2: 2018–2019	
	Boys (*n* = 2284)	Girls (*n* = 3679)	Boys (*n* = 2284)	Girls (*n* = 3679)
Demographic				
Age, y	12.9 ± 2.4	15.8 ± 2.9	15.8 ± 2.4	18.7 ± 2.9
Currently married	0.0	45.9	2.6	52.6
Currently pregnant	0.0	21.0	0.0	16.1
Belongs to Hindu religion	85.8	80.2	85.8	80.5
Belongs to a backward caste	82.4	83.4	82.0	84.0
Wealth index: poorest	14.0	14.7	14.0	14.7
Lives in urban area	15.3	13.0	15.3	13.0
Education, y	5.9 ± 2.7	6.6 ± 3.8	8.2 ± 2.7	7.8 ± 4.1
Currently attending school	91.1	51.2	75.2	32.2
Mother's education, y	2.4 ± 4.2	1.8 ± 3.6	2.4 ± 4.2	1.8 ± 3.6
Environmental				
Improved source of drinking water	96.8	97.8	98.4	97.6
Improved latrine facility at home	30.1	30.3	58.3	51.9
In government school^2^	54.9	67.0	56.7	67.9
In private school^2^	45.1	32.7	43.2	31.7
Exposure to mass media	35.5	20.1	60.0	38.8
Ever used social media	8.9	2.6	50.3	20.8

1 Values are means ± SDs for continuous variables or percentages for categorical variables.

2 Percentages for type of school are among the subsample currently attending school.

### Reading and math proficiency

**FIGURE 1:**
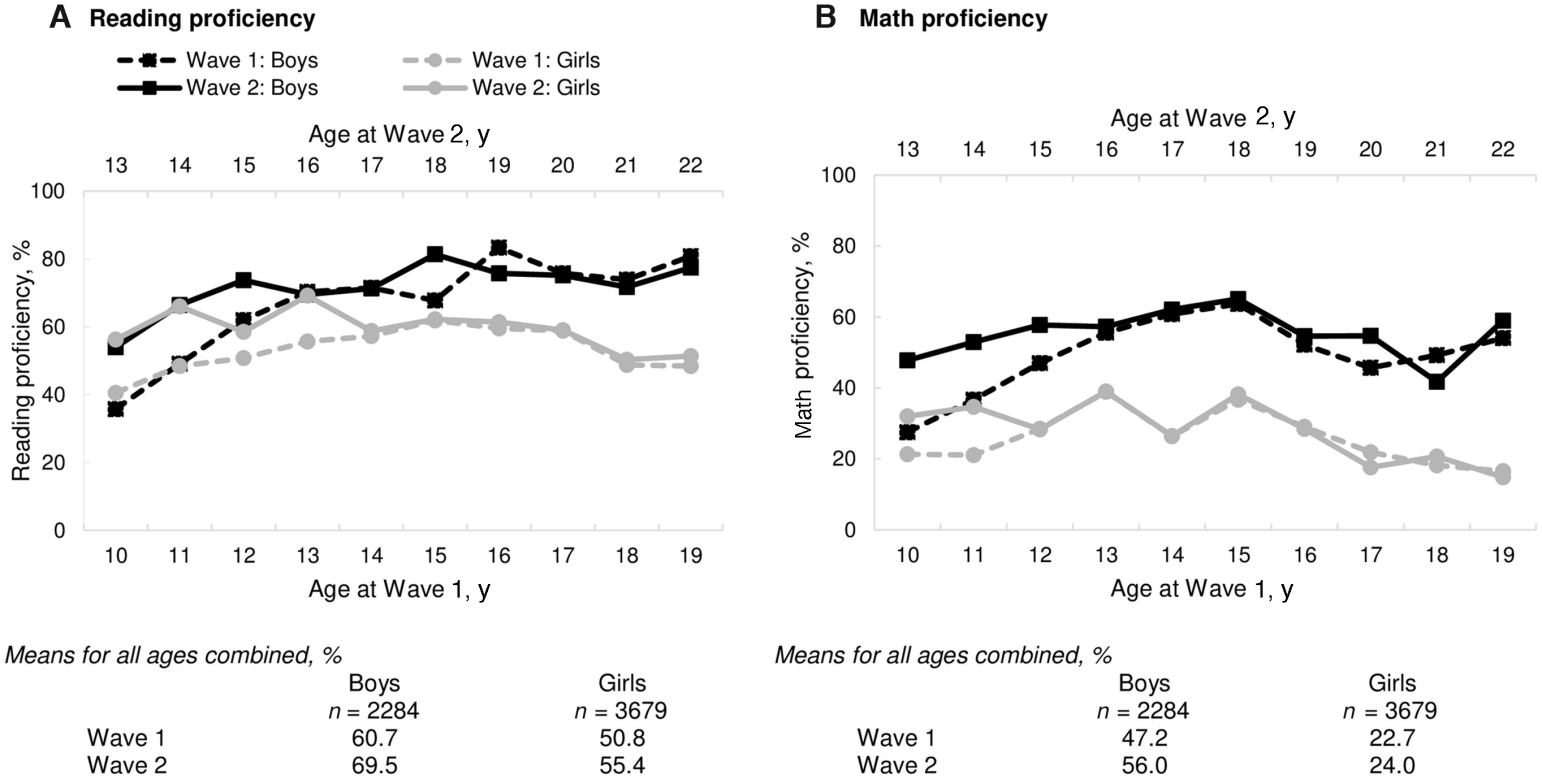
Reading (A) and math proficiency (B) in Indian adolescents by age, gender, and survey wave, Understanding the Lives of Adolescents and Young Adults (UDAYA) data 2015–2016 and 2018–2019. Reading proficiency: ability to read a story in the Hindi language; math proficiency: ability to solve a 3-digit division problem.

Overall, reading and math proficiency were low. At wave 1, on average, only 61% of boys and 51% of girls were able to read a story (**Figure 1**A). Even by 19 y of age, about one-fifth of boys and half of girls could not read a story that children in standard II (7–8 y of age) should be able to read. Overall, reading proficiency improved for both boys (9 pp) and girls (5 pp) at the follow-up survey. Only 47% of boys and 23% of girls were able to solve a division problem in wave 1 (Figure 1B). Math proficiency showed improvement over time for boys (9 pp), but not for girls. The gender inequality in reading and math proficiency was more pronounced for math than for reading in both survey waves.

### Prevalence of anemia, thinness, and stunting

**FIGURE 2:**
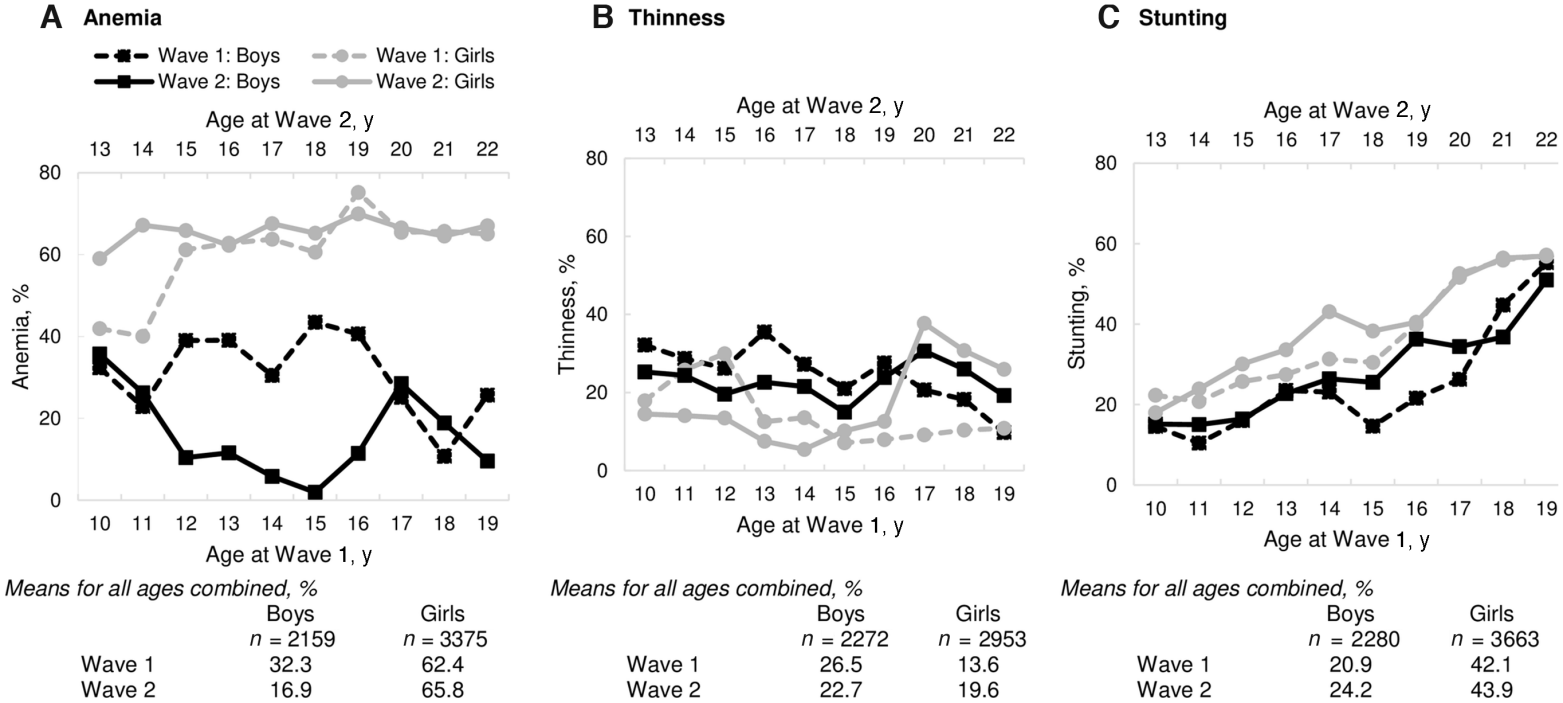
Prevalence of anemia (A), thinness (B), and stunting (C) in Indian adolescents by age, gender, and survey wave, Understanding the Lives of Adolescents and Young Adults (UDAYA) data 2015–2016 and 2018–2019.

Anemia was higher for girls than for boys at all ages from 10 to 22 y, with an overall prevalence of 62% for girls compared with 32% for boys in wave 1 of the survey (**Figure 2**A). Anemia decreased with increasing age in boys but remained consistent for girls across ages 12–22 y. Between the 2 survey waves, anemia reduced by nearly half for boys (from 32% to 17% for all ages combined), but slightly increased for girls (from 62% to 66%). In contrast, overall thinness was lower among girls than among boys (14% compared with 27% in wave 1, 20% compared with 23% in wave 2) (Figure 2B). The prevalence of thinness was similar for adolescents of different ages in both waves and increased after the age of 15 y for girls. Stunting was higher among girls than among boys (42% compared with 21% in wave 1, 44% compared with 24% in wave 2) and higher among older adolescents in both survey waves (Figure 2C).

**FIGURE 3:**
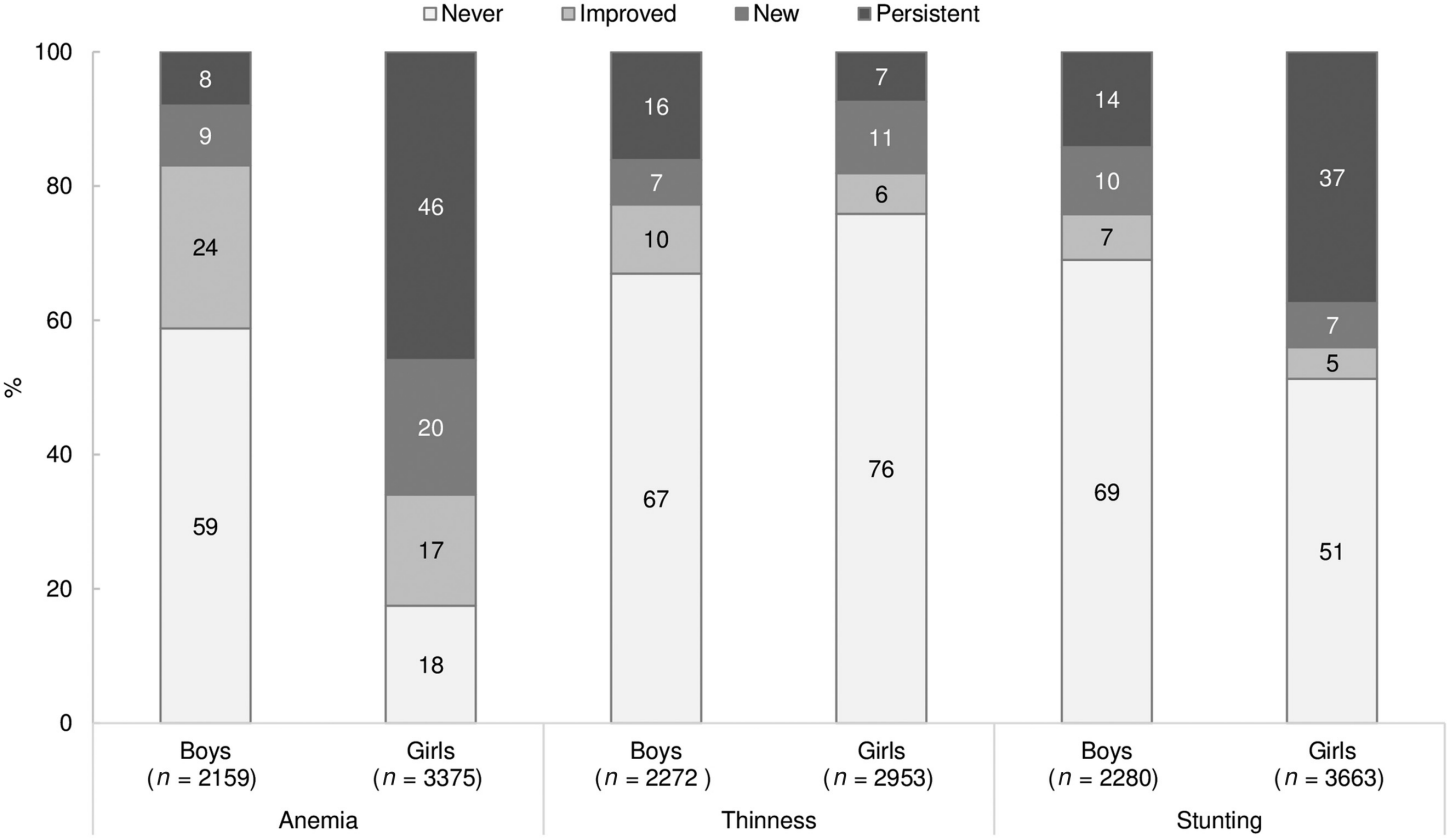
Status of anemia, thinness, and stunting among adolescents over time, Understanding the Lives of Adolescents and Young Adults (UDAYA) data 2015–2019. The categories were defined as follows (using anemia as an example): never (nonanemic in both waves), new (anemic only in wave 2), improved (anemic only in wave 1), persistent (anemic in both waves). Similar categories were created for thinness and stunting.

Improvement in anemia and anthropometry was 1.4–1.7 times higher among boys than among girls. For example, 24% of boys experienced improvements in anemia status compared with 17% of girls, 10% compared with 6% experienced improvements in thinness status, and 7% compared with 5% improved their stunting status (**Figure 3**). In contrast, persistent anemia was 5.8 times lower in boys than in girls (8% compared with 46%). New anemia was also more common in girls than in boys (20% compared with 9%). The prevalence of persistent thinness was higher in boys than in girls (16% compared with 7%), whereas persistent stunting was 2.6 times higher in girls than in boys (37% compared with 14%).

### Association between change in anemia/anthropometry over time and learning outcomes

Persistently anemic adolescents had statistically significantly lower learning skills than those who were not anemic in both waves, and this remained true after adjusting for confounding factors among boys (AOR: 0.26; 95% CI: 0.12, 0.59 for reading proficiency; AOR: 0.35; 95% CI: 0.16, 0.76 for math proficiency), but not among girls (**Table 2**, **Supplemental Table 3**). New anemia also predicted lower odds of reading proficiency among boys (AOR: 0.42; 95% CI: 0.19, 0.89). Boys whose anemia status improved between waves 1 and 2 had similar learning skills as boys who were not anemic in either wave.

**TABLE 2:** Association of changes in anemia, thinness, or stunting status with learning outcomes among adolescent boys and girls, Understanding the Lives of Adolescents and Young Adults (UDAYA) data 2015–2019^1^

	Reading proficiency				Math proficiency			
	Boys		Girls		Boys		Girls	
	Unadjusted model, OR (95% CI)	Adjusted model, AOR (95% CI)	Unadjusted model, OR (95% CI)	Adjusted model, AOR (95% CI)	Unadjusted model, OR (95% CI)	Adjusted model, AOR (95% CI)	Unadjusted model, OR (95% CI)	Adjusted model, AOR (95% CI)
Change in anemia prevalence over time								
Never (reference)	1	1	1	1	1	1	1	1
New	0.15*** (0.07, 0.36)	0.42* (0.19, 0.89)	0.69 (0.39, 1.21)	0.92 (0.53, 1.60)	0.51 (0.25, 1.05)	0.73 (0.36, 1.47)	1.22 (0.74, 2.01)	1.28 (0.77, 2.13)
Improved	0.56* (0.33, 0.94)	0.87 (0.52, 1.44)	0.70 (0.38, 1.29)	1.00 (0.55, 1.82)	0.77 (0.48, 1.25)	1.04 (0.65, 1.66)	0.77 (0.45, 1.31)	0.96 (0.55, 1.67)
Persistent	0.04*** (0.02, 0.12)	0.26*** (0.12, 0.59)	0.54* (0.33, 0.88)	0.95 (0.59, 1.54)	0.18*** (0.08, 0.39)	0.35** (0.16, 0.76)	0.94 (0.61, 1.45)	1.31 (0.84, 2.04)
Change in thinness prevalence over time								
Never (reference)	1	1	1	1	1	1	1	1
New	0.83 (0.36, 1.94)	0.76 (0.33, 1.78)	0.51* (0.28, 0.94)	1.12 (0.60, 2.10)	0.49 (0.23, 1.06)	0.57 (0.27, 1.21)	0.56* (0.33, 0.96)	1.23 (0.69, 2.17)
Improved	0.31** (0.15, 0.64)	0.77 (0.40, 1.47)	0.60 (0.27, 1.30)	0.92 (0.43, 1.96)	0.42** (0.22, 0.79)	0.65 (0.35, 1.20)	0.74 (0.37, 1.45)	0.68 (0.35, 1.35)
Persistent	0.16*** (0.08, 0.30)	0.37*** (0.21, 0.66)	1.80 (0.86, 3.79)	1.30 (0.63, 2.67)	0.19*** (0.11, 0.33)	0.27*** (0.16, 0.46)	0.92 (0.48, 1.74)	0.83 (0.44, 1.59)
Change in stunting prevalence over time								
Never (reference)	1	1	1	1	1	1	1	1
New	0.32** (0.15, 0.67)	0.38** (0.19, 0.75)	0.99 (0.51, 1.90)	1.78 (0.93, 3.37)	0.42** (0.22, 0.81)	0.52* (0.27, 0.98)	0.77 (0.43, 1.37)	1.12 (0.62, 2.01)
Improved	0.17*** (0.07, 0.43)	0.40* (0.17, 0.91)	0.50 (0.23, 1.11)	0.79 (0.37, 1.69)	0.20*** (0.09, 0.45)	0.33** (0.15, 0.73)	0.66 (0.33, 1.33)	0.81 (0.40, 1.63)
Persistent	0.23*** (0.12, 0.44)	0.37** (0.19, 0.69)	0.14*** (0.09, 0.21)	0.46*** (0.32, 0.68)	0.19*** (0.10, 0.34)	0.29*** (0.16, 0.53)	0.19*** (0.13, 0.26)	0.46*** (0.32, 0.66)

1 The categories were defined as follows, using anemia as an example: never (nonanemic in both waves), new (anemic only in wave 2), improved (anemic only in wave 1), persistent (anemic in both waves). Similar categories were created for thinness and stunting. Values were estimated using multilevel multivariate mixed-effect models adjusted for survey wave; respondent's age, marital status, birth history for girls (currently pregnant or ever gave birth), number of siblings, whether attending school, type of school studied in, exposure to mass media, use of any social media platform; mother's education attainment; and household's place of residence (urban/rural), wealth status, religion, caste, access to improved toilet facility, and their use of an improved source of drinking water. AOR, adjusted odds ratio.

*P < 0.05, **P < 0.01, ***P < 0.001. Significant difference from reference category "Never."

Among boys, persistent thinness was negatively associated with both reading (AOR: 0.37; 95% CI: 0.21, 0.66) and math proficiency (AOR: 0.27; 95% CI: 0.16, 0.46) (Table 2, **Supplemental Table 4**). Boys who had new thinness or whose thinness status improved showed no difference in reading or math proficiency from those who were not thin in either wave. Girls who experienced new thinness had lower reading and math proficiency than those who were never thin in unadjusted models, but these associations did not hold after adjustment for potentially confounding factors.

Stunting at any point—either new, improved, or persistent—predicted lower odds of being able to read a story (AOR: 0.37–0.40) in boys (Table 2, **Supplemental Table 5**). Stunting also predicted poorer math ability, with the negative association strengthening from new (AOR: 0.52; 95% CI: 0.27, 0.98) to improved (AOR: 0.33; 95% CI: 0.15, 0.73) to persistent (AOR: 0.29; 95% CI: 0.16, 0.53) stunting. Persistent stunting also predicted poorer reading and math proficiency in girls, but girls whose stunting status improved showed statistically similar reading and math proficiency as girls who were not stunted in both waves.

## Discussion

Using panel data following adolescents in northern India for 3 y, we estimated the association between changes in anthropometry or anemia status and learning outcomes. We found that persistent anemia, thinness, and stunting were associated with lower reading and math proficiency in boys, whereas persistent stunting was associated with poorer learning skills in girls. Boys who became anemic or stunted over this period were also less likely to be able to solve division problems than boys who were nonanemic or nonstunted at both time points. Boys whose anemia or thinness status improved and girls whose linear growth status improved showed comparable reading and math proficiency with those who were not anemic, stunted, and/or thin at either survey time point.

The negative association between anthropometric failure or anemia and learning outcomes during adolescence in our study is consistent with other studies that have reported negative associations between early-life insults and developmental outcomes in future years such as Young Lives (13, 15, 29–32), the Malnutrition and Enteric Disease Study (MAL-ED) multinational studies (33), the Consortium of Health Orientated Research in Transitioning Societies (COHORTS) (34), and other studies in low- and middle-income countries (35–45). Children who experienced stunting in the first 1000 d of life had poorer development of language and motor skills at 2 y of age in samples from Burkina Faso, Ghana, and Malawi (37); lower cognitive development at 5 y of age in multiple countries (33); and lower intelligence quotient (IQ) at 8–11 y of age in Peru and the Philippines (35, 36). Weight faltering in the first 9 mo of life was associated with persisting deficits in IQ at 8 y of age in a cohort from the United Kingdom (38) and weight gain during the first 2 y of life was strongly associated with schooling in adulthood in a multicountry analysis (34). Studies have also reported associations between anemia during infancy and poorer cognition, school achievement, or behavioral problems in middle childhood (46) and adolescence (47).

We found that boys whose anemia or thinness status improved and girls whose linear growth improved performed as well on reading and math tests as those with “normal” indicators at both survey time points. Findings from multinational studies have shown that children whose stunting status improved at 5, 6, and 8 y of age performed better in cognitive tests than those who remained stunted throughout or performed similarly to those who were never stunted (13, 30, 39). Similarly, weight gain in underweight Japanese children aged 12–13 y had a positive effect on their academic performance, independently of socioeconomic and lifestyle factors (48). A randomized controlled trial in Indian adolescent girls and boys found that consumption of high iron-biofortified pearl millet during school meals for 6 mo corrected iron deficiency and improved performance on computerized tests of attention and memory (18). Another iron intervention in Indian girls found that Hb gain after iron and folic acid supplementation for 1 y was associated with improved cognitive ability (17). Whereas anthropometric failure likely reflects failure in a range of factors over time, there is a clearly established mechanism for iron deficiency anemia being a cause of suboptimal brain and behavioral outcomes (49). Taken together, the evidence makes a compelling case for promoting nutrition interventions, or interventions that improve living conditions and lead to improved nutrition, throughout the first 2 decades of life to achieve optimal learning outcomes.

Associations between change in anthropometry or anemia and learning outcomes differed by gender. Persistent anemia and thinness were negatively associated with learning outcomes in boys but not in girls, apart from persistent anemia showing a negative association with reading ability in girls. We also found that gender inequalities in learning outcomes increased with age, similar to previous studies (50, 51). Lower reading and math proficiency in girls than in boys could be explained by son preference (52) or cultural expectations for girls to drop out of school to perform household chores and marry at a young age (53–55). Another gender difference was that boys whose stunting status improved did not achieve similar learning abilities as their peers who were not stunted during adolescence, whereas girls whose stunting status improved did. Because boys are biologically more vulnerable to morbidity during their early years than girls, the interaction of early-life stunting and morbidity could have had a significant impact on their cognitive development, preventing them from catching up in their academic performance (56, 57). Further investigation into gender differences in the timing of insults to anthropometry and learning improvement is needed.

The associations identified in this study should be interpreted in a broader learning and living environment. Recent child development literature suggests that growth faltering is a marker of a poor environment and is not always a direct cause of poor cognitive development (58–60). At the same time, being physically smaller limits one's ability to interact with one's environment and acquire knowledge and skills (60). Caregivers or teachers may also knowingly or unknowingly treat smaller children differently and challenge them less than their peers. Thus, in terms of achieving optimal learning outcomes, interventions must address environmental and social barriers. A systematic review and meta-analysis of 75 intervention studies involving children aged 0–5 y found smaller effects of nutritional supplementation interventions on learning than for nurturing and stimulation interventions (58). Further research in different contexts is needed to assess the relative benefits of nutritional and nonnutritional interventions on learning outcomes in adolescents.

Strengths of our analysis include the large and representative longitudinal data on adolescents from 2 populous Indian states. These unique data provide rich information on multiple facets of the adolescent transition to adulthood, including learning outcomes, nutritional status, and various socioeconomic, demographic, and environmental factors. Using multilevel mixed-effects logistic regression models, we were able to track changes in physical growth/anemia status and examine how these changes related to reading and math proficiency 3 y later. By analyzing boys and girls separately, we have identified gender gaps in the prevalence of poor physical growth or anemia and poor learning skills. However, some data limitations must be noted. First, information on the quality of teaching was not available and information on the quality of schools could not be used. Given our interest in considering both in-school and out-of-school adolescents, we could not control for variables only available for in-school adolescents such as school-based programs and school infrastructure. Second, data on physical growth and anemia before adolescence were not available. Although it may be reasonable to assume correlations between preadolescent and adolescent growth and anemia statuses in this high-poverty population with limited opportunities for upward mobility (61), we are not able to confirm this hypothesis with the available data. Third, studying changes in linear growth during adolescence is complicated by growth spurts, which may occur at different times for the studied population and the “healthy" reference population used to generate HAZs (62). Our findings that relate changes in height-for-age to learning outcomes should therefore be interpreted with caution.

Given the demonstrated associations between physical growth, anemia, and learning outcomes during adolescence, investments in nutrition and human capital should cover late childhood and adolescence in addition to the first 1000 d. The recent *Lancet* series on adolescent health highlights the roles of nutrition in late childhood and early adolescence on the timing and pattern of puberty, physical growth, neurodevelopment, immunity, and risk of noncommunicable diseases in later life (63). The series also calls for multifaceted actions at local and national levels across sectors (education, health, food systems, social protection, and digital media) and tailoring of nutrition interventions to local contexts (64). To address adolescent well-being in India, several programs have been implemented under various ministries (65). Examples include the Adolescent Girls Scheme or “Kishori Shakti Yojana,” the Rajiv Gandhi Scheme for Empowerment of Adolescent Girls, the Balika Samridhi Yojana, and the Rashtriya Kishor Swasthya Karyakram. In 2018, adolescent nutrition in India received renewed political and program focus under the Prime Minister's Overarching Scheme for Holistic Nourishment (POSHAN) Abhiyaan and Anaemia Free India. Collectively, the findings to date underscore the importance of continued investment in adolescent-focused programs and research to better understand how to optimize program impacts for both girls and boys.

With <10 y remaining to achieve the Sustainable Development Goals of ending all forms of malnutrition and achieving universal literacy and numeracy, targeted and integrated health-nutrition-cognition interventions covering the first 8000 d of life are needed. Our study found that anemia and anthropometric failure were associated with poor learning outcomes and these effects were more pronounced among boys than among girls. Adolescents whose anthropometry or anemia status improved in the 3-y period between the 2 surveys showed similar learning abilities as their peers who were not undernourished at either survey. Beyond interventions directly targeting learning, efforts to provide equal and adequate learning opportunities to both boys and girls, as well as interventions that enhance standards of living and contribute to improvements in anthropometry and anemia, could help to support learning outcomes in adolescents.

## Supplementary Material

nqac028_Supplemental_File

## Data Availability

Data described in the article, codebook, and analytic code will be made available upon request.

## Notes

The primary data collection for the Understanding the Lives of Adolescents and Young Adults (UDAYA) survey was supported by the Bill & Melinda Gates Foundation and the David and Lucile Packard Foundation through a grant to the Population Council. This secondary analysis was supported by Bill & Melinda Gates Foundation grant OPP1150189 (to PM) through the Partnerships and Opportunities to Strengthen and Harmonize Actions for Nutrition in India (POSHAN), led by the International Food Policy Research Institute. The study sponsor had no role in the study design, data collection, data analysis, data interpretation, report writing, or decision to submit the manuscript for publication.

Supplemental Figure 1 and Supplemental Tables 1–5 are available from the “Supplementary data” link in the online posting of the article and from the same link in the online table of contents at https://academic.oup.com/ajcn/.

Abbreviations used: AOR, adjusted odds ratio; ASER, Annual Status of Education Report; BMIZ, BMI-for-age *z*-score; HAZ, height-for-age *z*-score; Hb, hemoglobin; IQ, intelligence quotient; pp, percentage points; PSU, primary sampling unit; UDAYA, Understanding the Lives of Adolescents and Young Adults.
