# Supplementary material for: Changes in anemia and anthropometry during adolescence predict learning outcomes: findings from a 3-year longitudinal study in India
Source: Am J Clin Nutr. 2022 Feb 4;115(6):1549–58. doi: 10.1093/ajcn/nqac028 (PMC9170477; doi:10.1093/ajcn/nqac028)
Supplement: nqac028_Supplemental_File [file nqac028_supplemental_file.docx]

**Table of contents**

| **S.No.** | **Table/Figures** | **Description** |
| --- | --- | --- |
| 1 | Supplementary Figure 1 | Sample flow |
| 2 | Supplementary Table 1 | Variable definition |
| 3 | Supplementary Table 2 | Distribution of demographic, health, social and environmental characteristics of Indian adolescents in the analytical and non-analytical sample, UDAYA data 2015-2016 |
| 4 | Supplementary Table 3 | Association between changes in anemia during adolescence and learning outcomes among adolescent boys and girls |
| 5 | Supplementary Table 4 | Association between changes in thinness status during adolescence and learning outcomes among adolescent boys and girls |
| 6 | Supplementary Table 5 | Association between changes in stunting status during adolescence and learning outcomes among adolescent boys and girls |

**Supplementary Figure 1: Sample flow**


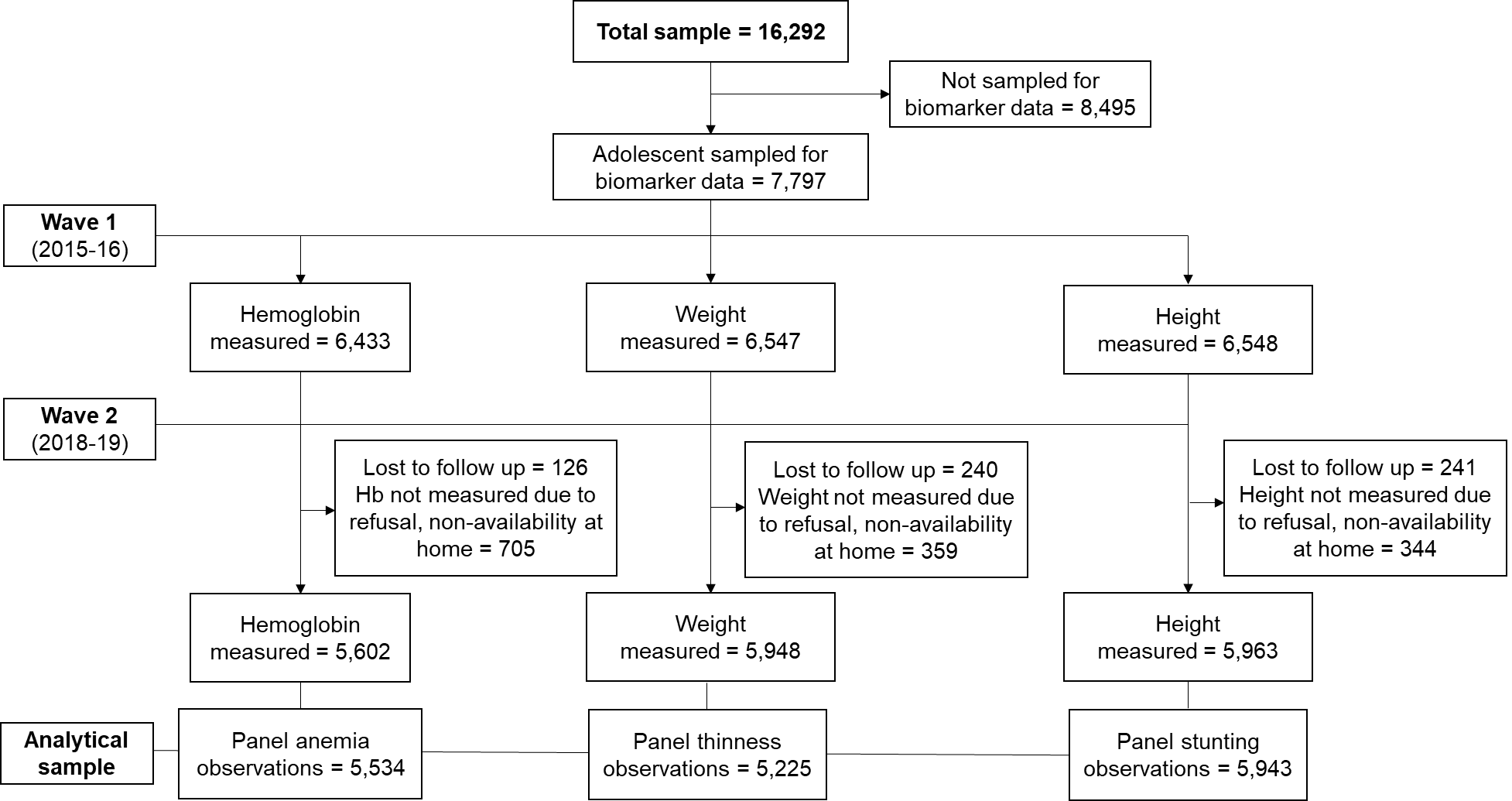


**Supplementary Table 1: Variable definition**

| **Indicator** | **Definition** |
| --- | --- |
| **Outcomes** |  |
| Reading proficiency | Respondent’s ability to read at story level (Hindi language) assessed using ASER tools [1]. |
| Math proficiency | Respondent’s ability to solve three-digit division problem assessed using ASER tools [1]. |
| **Predictors** |  |
| Anemia | The dummy of any anemia presence (mild, moderate or severe) was constructed using [WHO guidelines](https://www.who.int/vmnis/indicators/haemoglobin.pdf) [2].   - Adolescents aged 11 years or younger are considered anaemic if their Hb level <115 g/l. - Adolescents aged 12-14 years and non-pregnant women (≥15 years) were considered anaemic if their Hb level <120 g/l. - Pregnant girls (≥15 years) are considered anaemic if their Hb level <110 g/l.   Boys (≥15 years) were considered anaemic if their Hb level <130 g/l. |
| Never anemic | Non-anemic adolescents in both wave 1 and wave 2 of the survey |
| New anemia | Adolescents anemic in wave 2 but not in wave 1 of the survey |
| Recovered anemia | Adolescents anemic in wave 1 but not in wave 2 of the survey |
| Persistent anemia | Adolescents anemic in both wave 1 and wave 2 of the survey |
| Thinness | For adolescents in aged 10-19 years age and sex specific BMI-for-age z score was computed as per [WHO criteria](http://www.euro.who.int/en/health-topics/disease-prevention/nutrition/a-healthy-lifestyle/body-mass-index-bmi), 2007 [3]. For those aged 20-23 years, BMI was calculated as weight (kg)/height (m^2^). Underweight was defined as BMIZ <-2 for those aged 10-19 years or BMI <18.5 for 20-23 years old.  Currently pregnant adolescents and those who had a birth in two months before survey were excluded. |
| Never thin | Adolescents with no thinness in both wave 1 and wave 2 of the survey |
| New thinness | Adolescents with thinness in wave 2 but not in wave 1 of the survey |
| Recovered thinness | Adolescents with thinness in wave 1 but not in wave 2 of the survey |
| Persistent thinness | Adolescents with thinness in both wave 1 and wave 2 of the survey |
| Stunting | Height-for-age Z-score (HAZ) was calculated using zanthro package in State 16. For the sample aged 10-19 years, HAZ was computed using WHO criteria [3]. For the sample aged 20-23 years, HAZ was computed using UK criteria [4]. Stunting was defined as HAZ< -2SD. |
| Never stunted | Adolescents with no stunting in both wave 1 and wave 2 of the survey |
| New stunting | Adolescents with stunting in wave 2 but not in wave 1 of the survey |
| Recovered stunting | Adolescents with stunting in wave 1 but not in wave 2 of the survey |
| Persistent stunting | Adolescents with stunting in both wave 1 and wave 2 of the survey |
| **Confounding factors** |  |
| Age, years | Age of respondent in continuous year |
| Currently married | Respondent is married - both with and without gauna. Gauna is a northern Indian custom and ceremony associated with the consummation of marriage in case of child marriage. The ceremony takes place several years after marriage. Before the ceremony, the bride stays at her natal home. Conjugal life begins only after gauna. |
| Currently pregnant | Respondent or his spouse was pregnant during survey |
| Lives in urban area | Urban residence. Here, reference category is rural residence. |
| Wealth quintile | Wealth index was constructed by allocating the following scores to a household’s reported assets or amenities:   1. Type of house: 2 for pucca; 1 for semi-pucca; 0 for kachcha. 2. Agricultural land owned: 5 for more than 10 acres; 4 for 5.1–10 acres; 3 for 2.6–5 acres; 2 for less than 2.6 acres, or if the household owns some land but does not know how much; 0 for no land. 3. Irrigated land owned: 1 for any irrigated land; 0 for no land. 4. Access to toilet facility: 4 for own flush toilet; 2 for shared flush toilet or own pit toilet; 1 for shared pit toilet or other types of toilet; 0 for no toilet facility. 5. Cooking fuel used: 2 for liquid petroleum gas, electricity, or biogas; 1 for kerosene, wood, crop residue, dung cakes, coal, or charcoal; 0 for other types of cooking fuel, for example, straw, shrubs, or grass. 6. Access to drinking water facility: 4 for own piped water, hand-pump, or covered well; 3 for own open well; 2 for public or shared piped water, hand-pump, or covered well; 1 for public or shared open well; 0 for other sources of drinking water, for example, surface water, water tanker/truck, or rainwater. 7. Access to electricity: 3 for electricity; 0 for no electricity. 8. Ownership of household assets: 4 for car or truck; 3 each for motorcycle or scooter, refrigerator, computer/laptop, telephone (landline or mobile), colour television; 2 each for bicycle, electric fan, sewing machine, thresher, water pump, animal-drawn cart; 1 for watch or clock; 0 for each of the above items that the household does not possess.   Constructed index ranged from 0 to 57. Households were then ranked according to the index score. This ranked sample was divided into quintiles with the first quintile representing households of the lowest (poorest) wealth status and the fifth quintile representing households with the highest (wealthiest) status. Wealth quintiles were developed at the state level based on the weighted sample for the whole state. |
| Belongs to a backward caste | Respondent belongs to a backward caste which includes scheduled caste, scheduled tribe and other backward class. Here, reference category refers to respondents who belong to a general caste. |
| Belongs to Hindu religion | Respondent belongs to Hindu religion. Here, reference category refers to respondents who belong to other religions including Muslim, Christian, Sikh, Buddhist and Jain. |
| Currently attending school | Respondent is currently going to school |
| Mother’s education | Highest level of schooling completed by the mother (in years). |
| School type | A dummy of school type is created with two levels- studies or studied in a government school or in a private school. |
| Improved source of drinking water | Availability of piped water on premises (piped household water connection located inside respondent’s dwelling, plot or yard) or other improved drinking water sources such as public taps or standpipes, tube wells or boreholes, protected dug wells, protected springs and rainwater collection. |
| Improved latrine facility at home | Availability of own flush toilet or own pit toilet. The indicator is constructed based on [WHO’s definition](https://www.who.int/data/gho/data/indicators/indicator-details/GHO/population-using-improved-sanitation-facilities-(-)) of an improved latrine facility. |
| Exposure to mass media | Out of the five sources (TV, radio, newspaper/magazine/books, own mobile, internet) respondent was exposed either every day or at least once a week to the content on any of the three mass media sources |
| Ever used social media | Respondent ever used any social media platform such as Facebook, Twitter |

**Supplementary Table 2: Distribution of demographic, health, social and environmental characteristics of Indian adolescents in the analytical and non-analytical sample in Wave 1, UDAYA, 2015-2016^1^**

|  | **Boys** | | **Girls** | |
| --- | --- | --- | --- | --- |
|  | **Analytical biomarker sample** | **Non-analytical biomarker sample** | **Analytical biomarker sample** | **Non-analytical biomarker sample** |
|  | (N = 2,284) | (N = 388) | (N = 3,679) | (N = 1,446) |
| **Demographic** |  |  |  |  |
| Age, years | 12.9 ± 2.4 | 14.4 ± 2.7 | 15.8 ± 2.9 | 17.3 ± 2.0 |
| Currently married | 0.0 | 0.0 | 45.9 | 74.4 |
| Currently pregnant | NA | NA | 21.0 | 28.8 |
| Belongs to Hindu religion | 85.8 | 80.1 | 80.2 | 81.2 |
| Belongs to a backward caste | 82.4 | 81.4 | 83.4 | 86.0 |
| Wealth index: poorest | 14.0 | 12.2 | 14.7 | 13.3 |
| Lives in urban area | 15.3 | 21.4 | 13.0 | 14.5 |
| Education, completed years | 5.9 ± 2.7 | 6.9 ± 3.0 | 6.6 ± 3.8 | 6.8 ± 4.1 |
| Currently attending school | 91.1 | 78.8 | 51.2 | 26.5 |
| Mother’s education, completed years | 2.4 ± 4.2 | 2.3 ± 4.0 | 1.8 ± 3.6 | 1.6 ± 3.3 |
| **Environmental** |  |  |  |  |
| Improved source of drinking water | 96.8 | 97.2 | 97.8 | 97.4 |
| Improved latrine facility at home | 30.1 | 36.8 | 30.3 | 29.5 |
| In government school | 54.9 | 53.2 | 67.0 | 73.5 |
| In private school | 45.1 | 46.8 | 32.7 | 26.5 |
| Exposure to mass media | 35.5 | 43.9 | 20.1 | 23.3 |
| Ever used social media | 8.9 | 14.5 | 2.6 | 1.9 |

^1^Among the subsample with anthropometric and/or biomarker data collected at wave 1 and did not provide data at wave 2

**Supplementary Table 3: Association between changes in anemia during adolescence and learning outcomes among adolescent boys and girls**

|  | **Reading proficiency** | | | | **Math proficiency** | | | |
| --- | --- | --- | --- | --- | --- | --- | --- | --- |
|  | ***Boys*** | | ***Girls*** | | ***Boys*** | | ***Girls*** | |
|  | **AOR** | **95% CI** | **AOR** | **95% CI** | **AOR** | **95% CI** | **AOR** | **95% CI** |
| Change in anemia prevalence over time^1^ |  |  |  |  |  |  |  |  |
| Never (reference) | 1 | -- | 1 | -- | 1 | -- | 1 | -- |
| New | 0.42^*^ | [0.19,0.89] | 0.92 | [0.53,1.60] | 0.73 | [0.36,1.47] | 1.28 | [0.77,2.13] |
| Improved | 0.87 | [0.52,1.44] | 1.00 | [0.55,1.82] | 1.04 | [0.65,1.66] | 0.96 | [0.55,1.67] |
| Persistent | 0.26^**^ | [0.12,0.59] | 0.95 | [0.59,1.54] | 0.35^**^ | [0.16,0.76] | 1.31 | [0.84,2.04] |
| Survey year: 2019 | 0.83 | [0.57,1.22] | 0.64^**^ | [0.46,0.87] | 1.86^***^ | [1.31,2.63] | 1.04 | [0.78,1.40] |
| Age of respondent | 1.61^***^ | [1.45,1.78] | 1.60^***^ | [1.47,1.75] | 1.19^***^ | [1.09,1.29] | 1.10* | [1.02,1.18] |
| Currently married | 0.32^*^ | [0.11,0.92] | 0.41^***^ | [0.24,0.70] | 0.53 | [0.18,1.58] | 0.57* | [0.33,0.97] |
| Currently pregnant or ever had birth |  |  | 0.44^***^ | [0.28,0.68] |  |  | 0.93 | [0.58,1.49] |
| Number of siblings | 0.90 | [0.71,1.14] | 0.97 | [0.81,1.15] | 0.93 | [0.75,1.16] | 0.90 | [0.76,1.06] |
| Currently attending school | 21.30^***^ | [12.84,35.31] | 8.20^***^ | [5.47,12.30] | 17.43^***^ | [10.81,28.10] | 5.24*** | [3.56,7.71] |
| Studied in private school/college | 1.59^*^ | [1.11,2.28] | 2.51^***^ | [1.74,3.62] | 0.73 | [0.52,1.01] | 1.01 | [0.74,1.39] |
| High exposure to mass media | 1.89^***^ | [1.31,2.72] | 1.92^***^ | [1.40,2.63] | 1.79^***^ | [1.27,2.53] | 2.28^***^ | [1.70,3.05] |
| Ever used any social media platform | 1.18 | [0.76,1.84] | 1.90^**^ | [1.20,3.02] | 0.93 | [0.63,1.37] | 0.94 | [0.64,1.37] |
| Mother's education: 1-7 years | 1.58 | [0.77,3.23] | 2.48^**^ | [1.42,4.33] | 1.42 | [0.73,2.75] | 3.21^***^ | [1.95,5.29] |
| 8-9 years | 4.69^***^ | [2.06,10.70] | 5.39^***^ | [2.74,10.61] | 5.20^***^ | [2.56,10.54] | 3.39^***^ | [1.94,5.93] |
| 10 years and above | 5.23^***^ | [2.14,12.77] | 17.30^***^ | [6.76,44.31] | 4.29^***^ | [2.14,8.61] | 13.50^***^ | [7.08,25.76] |
| Rural area | 0.89 | [0.50,1.58] | 0.95 | [0.58,1.54] | 1.97^*^ | [1.17,3.31] | 0.92 | [0.61,1.38] |
| Wealth quintile: Poor | 1.63 | [0.80,3.33] | 2.22^**^ | [1.21,4.06] | 1.58 | [0.78,3.18] | 1.94^*^ | [1.03,3.66] |
| Middle | 2.11^*^ | [1.04,4.30] | 4.43^***^ | [2.45,8.02] | 2.77^**^ | [1.39,5.53] | 3.22^***^ | [1.75,5.91] |
| Rich | 3.92^***^ | [1.81,8.46] | 9.88^***^ | [5.23,18.66] | 4.53^***^ | [2.18,9.42] | 5.06^***^ | [2.71,9.45] |
| Richest | 9.27^***^ | [3.73,23.03] | 19.46^***^ | [9.06,41.78] | 8.18^***^ | [3.58,18.66] | 8.21^***^ | [4.06,16.63] |
| Religion: Hindu | 2.27^*^ | [1.21,4.28] | 0.88 | [0.55,1.41] | 2.77^***^ | [1.53,5.02] | 2.96^***^ | [1.88,4.65] |
| Caste: SC/ST/OBC | 0.58 | [0.32,1.05] | 0.82 | [0.52,1.28] | 0.97 | [0.59,1.61] | 1.01 | [0.67,1.51] |
| Access to improved toilet facility | 0.87 | [0.59,1.28] | 1.25 | [0.92,1.71] | 1.42 | [0.99,2.03] | 0.89 | [0.66,1.21] |
| Improved source of drinking water | 2.54 | [0.92,6.97] | 1.18 | [0.46,3.03] | 1.00 | [0.37,2.72] | 1.74 | [0.72,4.20] |
| N | 4117.00 |  | 5462.00 |  | 4109.00 |  | 5421.00 |  |

^*^ *p* < 0.05, ^**^ *p* < 0.01, ^***^ *p* < 0.001

^1^ The categories were defined as follows: never (non-anemic in both waves), new (anemic only in wave 2), improved (anemic only in wave 1), persistent (anemic in both waves). Values are adjusted odds ratio (OR) and 95% confidence intervals (CI). Estimated using multilevel multivariate mixed-effect models adjusted for survey wave, respondent’s age, marital status, birth history for girls (currently pregnant or ever gave birth), number of siblings, whether attending school, type of school studied in, exposure to mass media, use of any social media platform; mother’s education attainment; and household’s place of residence (urban/rural), wealth status, religion, caste, access to improved toilet facility, and their use of improved source of drinking water.

**Supplementary Table 4: Association between changes in thinness status during adolescence and learning outcomes among adolescent boys and girls**

|  | **Reading proficiency** | | | | **Math proficiency** | | | | |  |
| --- | --- | --- | --- | --- | --- | --- | --- | --- | --- | --- |
|  | ***Boys*** | | ***Girls*** | | ***Boys*** | | ***Girls*** | | |  |
|  | **AOR** | **95% CI** | **AOR** | **95% CI** | **AOR** | **95% CI** | | **AOR** | **95% CI** | |
| Change in thinness prevalence over time^1^ |  |  |  |  |  |  | |  |  | |
| Never (reference) | 1 | -- | 1 | -- | 1 | -- | | 1 | -- | |
| New thinness | 0.76 | [0.33,1.78] | 1.12 | [0.60,2.10] | 0.57 | [0.27,1.21] | | 1.23 | [0.69,2.17] | |
| Recovered thinness | 0.77 | [0.40,1.47] | 0.92 | [0.43,1.96] | 0.65 | [0.35,1.20] | | 0.68 | [0.35,1.35] | |
| Persistent thinness | 0.37^***^ | [0.21,0.66] | 1.30 | [0.63,2.67] | 0.27^***^ | [0.16,0.46] | | 0.83 | [0.44,1.59] | |
| Survey year: 2019 | 0.83 | [0.57,1.20] | 0.61^**^ | [0.44,0.86] | 1.66^**^ | [1.19,2.31] | | 1.06 | [0.78,1.44] | |
| Age of respondent | 1.67^***^ | [1.51,1.84] | 1.69^***^ | [1.54,1.86] | 1.24^***^ | [1.14,1.34] | | 1.12^**^ | [1.04,1.20] | |
| Currently married | 0.25^**^ | [0.09,0.70] | 0.39^**^ | [0.22,0.69] | 0.43 | [0.14,1.28] | | 0.42^**^ | [0.23,0.74] | |
| Currently pregnant or ever had birth |  |  | 0.39^***^ | [0.23,0.67] |  |  | | 0.95 | [0.54,1.69] | |
| Number of siblings | 0.94 | [0.75,1.18] | 0.97 | [0.80,1.19] | 0.97 | [0.79,1.19] | | 0.91 | [0.76,1.09] | |
| Currently attending school | 21.95^***^ | [13.45,35.84] | 9.48^***^ | [6.22,14.47] | 16.47^***^ | [10.38,26.13] | | 5.06^***^ | [3.41,7.52] | |
| Studied in private school/college | 1.64^**^ | [1.16,2.33] | 2.54^***^ | [1.73,3.74] | 0.69^*^ | [0.50,0.95] | | 0.92 | [0.66,1.27] | |
| High exposure to mass media | 2.07^***^ | [1.46,2.95] | 1.84^***^ | [1.31,2.60] | 1.72^**^ | [1.24,2.40] | | 2.36^***^ | [1.74,3.20] | |
| Ever used any social media platform | 1.18 | [0.77,1.81] | 1.82^*^ | [1.10,3.00] | 0.93 | [0.64,1.35] | | 0.75 | [0.50,1.12] | |
| Mother's education: 1-7 years | 1.59 | [0.79,3.20] | 3.22^***^ | [1.77,5.87] | 1.37 | [0.72,2.60] | | 3.25^***^ | [1.94,5.44] | |
| 8-9 years | 4.97^***^ | [2.21,11.16] | 7.58^***^ | [3.65,15.75] | 5.09^***^ | [2.55,10.15] | | 3.68^***^ | [2.07,6.55] | |
| 10 years and above | 4.08^***^ | [1.77,9.39] | 20.28^***^ | [7.74,53.14] | 3.64^***^ | [1.87,7.08] | | 13.59^***^ | [7.14,25.89] | |
| Rural area | 0.91 | [0.53,1.57] | 0.76 | [0.46,1.26] | 1.75^*^ | [1.06,2.88] | | 0.80 | [0.53,1.23] | |
| Wealth quintile: Poor | 1.62 | [0.81,3.25] | 2.17^*^ | [1.13,4.17] | 1.34 | [0.68,2.64] | | 1.90 | [0.97,3.75] | |
| Middle | 2.13^*^ | [1.07,4.24] | 4.03^***^ | [2.12,7.66] | 2.61^**^ | [1.34,5.10] | | 3.07^***^ | [1.60,5.88] | |
| Rich | 4.15^***^ | [1.97,8.73] | 9.63^***^ | [4.84,19.13] | 4.04^***^ | [2.00,8.18] | | 5.59^***^ | [2.89,10.84] | |
| Richest | 9.93^***^ | [4.10,24.03] | 18.78^***^ | [8.20,42.98] | 7.40^***^ | [3.33,16.41] | | 9.21^***^ | [4.37,19.42] | |
| Religion: Hindu | 2.30^**^ | [1.26,4.17] | 0.81 | [0.49,1.33] | 2.90^***^ | [1.65,5.09] | | 2.94^***^ | [1.84,4.69] | |
| Caste: SC/ST/OBC | 0.58 | [0.33,1.03] | 0.80 | [0.50,1.28] | 0.84 | [0.52,1.36] | | 1.03 | [0.68,1.55] | |
| Access to improved toilet facility | 0.85 | [0.58,1.24] | 1.13 | [0.81,1.59] | 1.37 | [0.97,1.94] | | 1.01 | [0.74,1.38] | |
| Improved source of drinking water | 2.39 | [0.90,6.37] | 0.82 | [0.30,2.22] | 1.02 | [0.39,2.67] | | 0.96 | [0.42,2.22] | |
| N | 4327.00 |  | 4938.00 |  | 4317.00 |  | | 4908.00 |  | |

^*^ *p* < 0.05, ^**^ *p* < 0.01, ^***^ *p* < 0.001

^1^ The categories were defined as follows: never (non-thin in both waves), new (thin only in wave 2), improved (thin only in wave 1), persistent (thin in both waves). Values are adjusted odds ratio (OR) and 95% confidence intervals (CI). Estimated using multilevel multivariate mixed-effect models adjusted for survey wave, respondent’s age, marital status, birth history for girls (currently pregnant or ever gave birth), number of siblings, whether attending school, type of school studied in, exposure to mass media, use of any social media platform; mother’s education attainment; and household’s place of residence (urban/rural), wealth status, religion, caste, access to improved toilet facility, and their use of improved source of drinking water.

**Supplementary Table 5: Association between changes in stunting status during adolescence and learning outcomes among adolescent boys and girls**

|  | **Reading proficiency** | | | | **Math proficiency** | | | | |
| --- | --- | --- | --- | --- | --- | --- | --- | --- | --- |
|  | ***Boys*** | | ***Girls*** | | | ***Boys*** | | ***Girls*** | |
|  | **AOR** | **95% CI** | **AOR** | **95% CI** | | **AOR** | **95% CI** | **AOR** | **95% CI** |
| Change in stunting prevalence over time^1^ |  |  |  |  | |  |  |  |  |
| Never (reference) | 1 | -- | 1 | -- | | 1 | -- | 1 | -- |
| New stunting | 0.38^**^ | [0.19,0.75] | 1.73 | [0.91,3.29] | | 0.52^*^ | [0.27,0.98] | 1.12 | [0.62,2.01] |
| Recovered stunting | 0.40^*^ | [0.17,0.91] | 0.76 | [0.35,1.62] | | 0.33^**^ | [0.15,0.73] | 0.81 | [0.40,1.63] |
| Persistent stunting | 0.37^**^ | [0.19,0.69] | 0.47^***^ | [0.32,0.69] | | 0.29^***^ | [0.16,0.53] | 0.46^***^ | [0.32,0.66] |
| Survey year: 2019 | 0.73 | [0.50,1.07] | 0.50^***^ | [0.37,0.68] | | 1.47^*^ | [1.05,2.06] | 0.92 | [0.69,1.22] |
| Age of respondent | 1.74^***^ | [1.57,1.93] | 1.71^***^ | [1.57,1.87] | | 1.29^***^ | [1.19,1.40] | 1.16^***^ | [1.07,1.24] |
| Currently married | 0.24^**^ | [0.09,0.69] | 0.41^***^ | [0.24,0.68] | | 0.43 | [0.14,1.27] | 0.56^*^ | [0.34,0.92] |
| Currently pregnant or ever had birth |  |  | 0.47^***^ | [0.31,0.71] | |  |  | 0.91 | [0.58,1.43] |
| Number of siblings | 0.94 | [0.75,1.18] | 1.00 | [0.85,1.19] | | 0.97 | [0.79,1.19] | 0.89 | [0.75,1.04] |
| Currently attending school | 21.57^***^ | [13.21,35.24] | 10.23^***^ | [6.90,15.17] | | 15.87^***^ | [10.01,25.17] | 5.46^***^ | [3.77,7.92] |
| Studied in private school/college | 1.65^**^ | [1.16,2.34] | 3.06^***^ | [2.15,4.37] | | 0.69^*^ | [0.50,0.95] | 0.95 | [0.70,1.30] |
| High exposure to mass media | 1.98^***^ | [1.39,2.82] | 2.07^***^ | [1.52,2.81] | | 1.67^**^ | [1.20,2.33] | 2.21^***^ | [1.66,2.93] |
| Ever used any social media platform | 1.18 | [0.77,1.82] | 1.76^*^ | [1.13,2.74] | | 0.92 | [0.63,1.34] | 0.94 | [0.65,1.36] |
| Mother's education: 1-7 years | 1.69 | [0.84,3.42] | 2.62^***^ | [1.54,4.47] | | 1.40 | [0.74,2.66] | 3.20^***^ | [1.98,5.15] |
| 8-9 years | 4.91^***^ | [2.17,11.10] | 6.53^***^ | [3.39,12.58] | | 4.90^***^ | [2.45,9.82] | 3.56^***^ | [2.07,6.13] |
| 10 years and above | 3.98^**^ | [1.72,9.23] | 21.10^***^ | [8.68,51.31] | | 3.47^***^ | [1.78,6.77] | 13.45^***^ | [7.27,24.90] |
| Rural area | 0.93 | [0.53,1.62] | 0.72 | [0.45,1.15] | | 1.86^*^ | [1.11,3.10] | 0.85 | [0.57,1.27] |
| Wealth quintile: Poor/Poorest | 1.58 | [0.79,3.17] | 0.23^***^ | [0.15,0.34] | | 1.34 | [0.68,2.65] | 1.99^*^ | [1.07,3.70] |
| Middle | 2.00^*^ | [1.01,4.00] |  |  | | 2.47^**^ | [1.26,4.83] | 3.09^***^ | [1.70,5.62] |
| Rich | 3.98^***^ | [1.89,8.39] |  |  | | 3.91^***^ | [1.92,7.93] | 5.05^***^ | [2.74,9.29] |
| Richest | 9.65^***^ | [3.97,23.43] |  |  | | 7.32^***^ | [3.29,16.29] | 7.21^***^ | [3.63,14.35] |
| Religion: Hindu | 2.47^**^ | [1.35,4.52] | 0.88 | [0.56,1.38] | | 3.04^***^ | [1.72,5.37] | 3.12^***^ | [2.01,4.84] |
| Caste: SC/ST/OBC | 0.59 | [0.33,1.04] | 0.76 | [0.49,1.18] | | 0.87 | [0.54,1.42] | 1.05 | [0.71,1.56] |
| Access to improved toilet facility | 0.83 | [0.57,1.22] | 1.51^**^ | [1.13,2.03] | | 1.35 | [0.96,1.91] | 0.89 | [0.67,1.19] |
| Improved source of drinking water | 2.42 | [0.91,6.43] | 1.14 | [0.46,2.85] | | 1.01 | [0.39,2.66] | 1.74 | [0.75,4.02] |
| N | 4329.00 |  | 5858.00 |  | | 4319.00 |  | 5810.00 |  |

^1^ The categories were defined as follows: never (non-stunted in both waves), new (stunted only in wave 2), improved (stunted only in wave 1), persistent (stunted in both waves). Values are odds ratio or adjusted odds ratio and 95% confidence intervals. OR: Odds ratio; AOR: Adjusted odds ratio; 95% confidence intervals. Estimated using multilevel multivariate mixed-effect models adjusted for survey wave, respondent’s age, marital status, birth history for girls (currently pregnant or ever gave birth), number of siblings, whether attending school, type of school studied in, exposure to mass media, use of any social media platform; mother’s education attainment; and household’s place of residence (urban/rural), wealth status, religion, caste, access to improved toilet facility, and their use of improved source of drinking water.

1. Ramaswami B, Wadhwa W. Survey Design and Precision of ASER Estimates. New Delhi: ASER Centre <http://img> asercentre org/docs/Aser% 20survey/Technical% 20Papers/precisionofaserestimates_ramaswami _wadhwa pdf*.*2010.

2. WHO. Haemoglobin concentrations for the diagnosis of anaemia and assessment of severity. Vitamin and Mineral Nutrition Information System. Geneva, World Health Organization (WHO/NMH/NHD/MNM/11.1). <http://www.who.int/vmnis/indicators/haemoglobin.pdf>. 2011.

3. de Onis M, Onyango AW, Borghi E, Siyam A, Nishida C, Siekmann J. Development of a WHO growth reference for school-aged children and adolescents. Bull World Health Organ 2007; 85:660-667.

4. Vidmar SI, Cole TJ, Pan H. Standardizing anthropometric measures in children and adolescents with functions for egen: Update. Stata Journal 2013; 13:366-378.
